# KAP Surveys and Dengue Control in Colombia: Disentangling the Effect of Sociodemographic Factors Using Multiple Correspondence Analysis

**DOI:** 10.1371/journal.pntd.0005016

**Published:** 2016-09-28

**Authors:** Diana Rocío Higuera-Mendieta, Sebastián Cortés-Corrales, Juliana Quintero, Catalina González-Uribe

**Affiliations:** 1 Eje de Salud Pública, Fundación Santa Fe de Bogotá, Bogotá, D.C., Colombia; 2 Department of Epidemiology, Bloomberg School of Public Health, Baltimore, Maryland, United States of America; 3 Department of Economics, University of Leicester, Leicester, United Kingdom; 4 School of Medicine, Universidad de los Andes, Bogotá, D.C., Colombia; Johns Hopkins Bloomberg School of Public Health, UNITED STATES

## Abstract

During the last few decades, several studies have analyzed and described knowledge, attitudes, and practices (KAP) of populations regarding dengue. However, few studies have applied geometric data analytic techniques to generate indices from KAP domains. Results of such analyses have not been used to determine the potential effects of sociodemographic variables on the levels of KAP. The objective was to determine the sociodemographic factors related to different levels of KAP regarding dengue in two hyper-endemic cities of Colombia, using a multiple correspondence analysis (MCA) technique. In the context of a cluster randomized trial, 3,998 households were surveyed in Arauca and Armenia between 2012 and 2013. To generate KAP indexes, we performed a MCA followed by a hierarchical cluster analysis to classify each score in different groups. A quantile regression for each of the score groups was conducted. KAP indexes explained 56.1%, 79.7%, and 83.2% of the variance, with means of 4.2, 1.4, and 3.2 and values that ranged from 1 to 7, 7 and 11, respectively. The highest values of the index denoted higher levels of knowledge and practices. The attitudes index did not show the same relationship and was excluded from the analysis. In the quantile regression, age (0.06; IC: 0.03, 0.09), years of education (0.14; IC: 0.06, 0.22), and history of dengue in the family (0.21; IC: 0.12, 0.31) were positively related to lower levels of knowledge regarding dengue. The effect of such factors gradually decreased or disappeared when knowledge was higher. The practices indexes did not evidence a correlation with sociodemographic variables. These results suggest that the transformation of categorical variables into a single index by the use of MCA is possible when analyzing knowledge and practices regarding dengue from KAP questionnaires. Additionally, the magnitude of the effect of socioeconomic variables on the knowledge scores varies according to the levels of knowledge, suggesting that other factors might be influencing higher levels of knowledge.

## Author Summary

Local vector control programs, as part of their routine activities, often use Knowledge Attitudes and Practices (KAP) surveys to guide their dengue control strategies. Usually, these questionnaires are extensive and result in a large amount of data that is difficult to analyze and summarize. This study uses an analytical approach to summarize the results of these types of questionnaires about dengue and subsequently assesses the effect of sociodemographic factors. Our results suggest that Multiple Correspondence Analysis is a useful statistical technique to summarize KAP survey information. Age and higher levels of education are related to more reported knowledge about dengue, but these effects are seen only among people in groups with low and middle knowledge of dengue, according to their KAP knowledge scores. Finally, when decisions about the family healthcare are made jointly by male and female members of the household, knowledge about the disease and means of transmission improves at all levels of knowledge (low, medium, high). Preventive practices regarding dengue, specifically against adult and immature forms of the mosquito that transmit the disease do not seem to be related to sociodemographic factors. This study provides an alternative way to more effectively analyze the results of KAP in a routine setting.

## Introduction

Dengue is an endemic or epidemic disease in countries located in the tropics [1], and approximately 40% of the world’s population is at risk of suffering dengue [2]. In Latin America, the number of cases has increased over the last three decades; it has grown from a low dengue-endemic to a hyper-endemic state in the majority of the region [3]. In 2010, Colombia faced the most important epidemic in the history of the country and reported the second-highest incidence in the Americas (157,203 cases). Since then, the country has reported the third and second-highest incidences on the continent [4,5].

Given the absence of a licensed and widely distributed tetravalent vaccine against dengue, integrated vector management (IVM) has played an important role in the control of the disease [3]. IVM is a process to optimize resources for vector control, and it has identified the assessment of local KAP as crucial in designing preventive interventions adapted to any context [6].

Since 2000, we found approximately 51 descriptive and analytical KAP studies regarding dengue in several countries, most of these papers are descriptive. The questionnaires used in such KAP studies have ranged between 13 [7] and 75 questions [8] and often result in a large number of dichotomous variables that are difficult to synthesize. Thirteen out of 48 revised studies have generated a single KAP variable through the construction of indexes based on what researchers consider a correct answer of each Knowledge, Attitude and Practice domain [8–19]. And only one has implemented a principal correspondence analysis (PCA) to summarize this information [20]. However, none of these fourteen studies with indexes has analyzed KAP data to identify the patterns of responses in each of its domains. And more importantly, the results from these analyses have not been used to determine the potential effects of sociodemographic variables on the levels of such indices of KAP (KAP index as a dependent variable).

KAP surveys are widely used in the broad context of public health, not only for research [21] but also for planning and intervention design [22,23]. Additionally, they are used to assess potential participation in prevention strategies of multiple diseases [22,23]; to evaluate the effectiveness of several public health interventions ranging from cardiovascular diseases [24,25] to tropical neglected diseases [26] and also to assess healthcare services in the hospital environment [27,28].

MCA is a descriptive method that allows the analysis of multiple categorical variables. It is widely used to generate assets and wealth indexes [29–32]. Recently, it has been used in the analysis of behavioral variables in HIV [32,33], healthy lifestyles [34,35], and hantavirus [36], amongst others. The generation of indices or scales from self-reported information has been an increasing need in social and public health sciences and new methods and theories have become increasingly popular during the last years, this is the case of latent variable analysis, factor analysis and item response theory amongst others [37]

In this context, KAP surveys provide a large amount of categorical data, and MCA allows the linking of separate sets of data efficiently for finding comparable trends between them [38]. The objective of this study was to determine the sociodemographic factors associated with certain KAP levels regarding dengue in two hyperendemic cities in Colombia using the MCA technique.

## Materials and Methods

### Study sites

KAP surveys were collected between December 2012 and April of 2013 in the cities of Colombia, Arauca and Armenia. Arauca, the capital city of the Arauca Department, has 85,994 inhabitants and is located on the border with Venezuela at 125 meters above mean sea level (MAMSL). It has a mean temperature of 30°C. Armenia, with 293,614 inhabitants, is the capital city of the Quindío Department and is located in the center of the country at 1,483 MAMSL, with temperatures ranging from 18°C to 29°C.

### Sample

We surveyed 3,998 households in the context of a cluster-randomized trial, following the same methods of Quintero et al [39] where a grid was overlapped in a satellite image of the two cities. Areas with empty land and non-residential zones were excluded. Of the remainder, 20 squares were randomly selected in each city, and 100 households were surveyed in each square beginning by the south-west corner of each square; the group of houses was called cluster. Personnel from the health authorities of both cities visited each household and invited the responsible adult available to participate in the study. In the case of absence two additional visits in different schedules were done. If contact was not possible after three visits the household was replaced by the contiguous household (Response rate: 99.95%).

### Instruments

The KAP questionnaire was based on a review of published studies using KAP surveys between 2001 and 2012 and on a review of the questionnaires provided by the authors of such studies. We developed our KAP survey using a combination of questions from various KAP questionnaires.

The new questionnaire was then piloted in a village near one of the study sites. After adaptation to the language in the local community, the 84-question survey was applied to each household using the mobile application e-mocha®, created by the Center for Clinical Global Health Education at the Johns Hopkins School of Medicine. The KAP survey had five sections: sociodemographic and gender decision-making information and knowledge, attitudes, and practices data (S1 File).

The sociodemographic section collected information about age, sex, education, income, number of persons per household, dwelling materials (floors and walls), migration, and access to public services. Given the already documented difficulties to capture household wealth with self-reported income [18], we used an additional measurement of socioeconomic strata that is used in Colombia to classify areas in the cities on a scale from 1 (lowest) to 6 (highest) and it is usually utilized to grant subsidies to the lowest-income population (strata 1) and to charge differential fees for public sanitation services [40,41]. The gender decision-making segment inquired about who decides about the health care of their own and others, daily expenses, large expenses, and household maintenance. The gender decision-making questions were extracted from the women’s module of the Demographic Health Survey [42].

Knowledge was defined as the understanding of a specific phenomenon, in this case, the means of transmission, symptoms, and means of prevention of dengue. An attitude refers to the organization of beliefs around a concept that predisposes to act in some specific manner. In this study, we asked for the severity of dengue and the repercussion for a case in the community, among others (e.g. Does a case of dengue in this community affects this household?”). Practices relate to a group of actions to ameliorate or trigger a specific outcome in health, in this case, actions toward the prevention of vector breeding sites [43,44].

### Ethics statement

All participants in this study provided oral and written informed consent before conducting the survey. The *Fundación Santa Fe de Bogotá’s* ethics committee, in compliance with all Colombian regulation governing the protection of human subjects, approved the protocol and the instruments of this study as recorded in the minutes of the meeting held on November 19, 2012.

### Statistical analysis

MCA was conducted to summarize the information of the categorical variables of the KAP survey into three scores (knowledge, attitudes, and practices) using the methods described by Kohn and Le Roux and Rouanet [38,45]. Unlike PCA, variables in this analysis do not need to follow a normal distribution, which makes MCA an appropriate approach for KAP variables, since most of them are categorical. We described all the dimensions extracted from the MCA; however, we chose the dimension with higher inertia for our analysis.

The first step of this process was the generation of a weight-per-answer option for each domain (knowledge, attitudes, and practices). To assign a score to each person according to their particular set of answers, a linear combination of the weights was done. For ease of interpretation, the scores were rescaled to be greater than or equal to 1, where 1 is the minimum value of the index.

These three scores were classified into different groups using hierarchical cluster analysis following the agglomerative method by average linkage presented by Kaufman and Rousseeuw [46,47]. Afterwards, we determined the number of groups through the Duda, Hart, and Stork stopping rules index [48]. Finally, a characterization of the most frequent answers in each group was done to determine the KAP profiles (low, medium, and high). In some cases, this process was not possible, given the heterogeneity of the answers.

A regression analysis was conducted to assess possible sociodemographic and gender determinants of the KAP scores. We considered fixed effects to account for the correlation within each cluster generated by unobservable variables. In the case of a non-normal distribution of the index, we did two-quantile regressions, considering fixed effects by clusters. We used STATA 13 for data depuration and analysis [49].

## Results

We surveyed 3,998 households (1,999 in each city). The average age of the respondents was 45 years old (std. dev. = 16), 74.81% were females, and the principal occupation reported was housewifery (44.20%), followed by working (40.22%). The mean years of education was 12 (std. dev. = 5), and the most frequent level of education was secondary education (25.01%). Of the total subjects, 65.59% reported income below two minimum salaries per month (one minimum monthly salary = 314.6 US Dollars).

Among the survey respondents, 44.32% were born in the same city where they currently live, 69.23% lived in the same city as their previous residence, 13.68% had moved from a rural area of the city to their current location, and 6.13% had lived in another municipality. The average number of inhabitants per household was four (std. dev. = 2) (Table 1). When inquiring about the number of members per family, we identified 14,702 individuals, 53.68% of them females. And 7.43% of the total respondents reported that they had been diagnosed with dengue at least once in their life

**Table 1:** Sociodemographic characteristics of population.

	Frequency/Mean	% / SD
Age:	45.19	15.85
Sex:		
Female	2991	74.81
Years of education	12.16	4.85
Occupation		
Worker	1,608	40.22
Housewifery	1,767	44.20
Student	200	5
Unemployed	130	3.25
Other	293	7.33
Socioeconomic Stratum		
1 (Lower low)	1,254	31.37
2 (Low)	1,430	35.77
3 (Upper low)	757	18.93
4 (Medium)	160	4
5 (Medium-high)	305	7.63
6 (High)	92	2.3
Income		
< 1MS	1228	30.72
1–2 MS	1394	34.87
>2 MS and ≤3 MS	361	9.03
>3 MS	181	6.36
Refuse to answer	834	20.86
Average number of inhabitants per household	3.6	1.64
Average number of women per household	1.95	1.11
Average number of workers per household	1.35	0.99
Average number of unemployed per household	0.094	0.33
Average number of inhabitants dedicated to housework per household	0.75	0.71
Years living in the same neighborhood	14.15	13.74
Years living in the same dwelling	12.37	12.91
Average number of dengue cases per household	0.27	0.62
Person who decides about: own healthcare		
Women	1,900	51.01
Men	454	12.19
Both	1,360	36.51
Nobody	11	0.3
Person who decide about: family healthcare		
Women	1,702	45.69
Men	359	9.64
Both	1,629	43.73
Not clear	35	0.94
Person who decide about: Household chores		
Women	2,013	54.04
Men	321	8.62
Both	1,386	37.21
Not clear	5	0.13
High expenses in the household		
Women	1,491	40.03
Men	512	13.74
Both	1,714	46.01
Not clear	8	0.21
Daily expenses in the household		
Women	1,970	52.89
Men	364	9.77
Both	1,381	37.07
Not clear	10	0.27

### Multiple correspondence analysis

**Fig 1:**
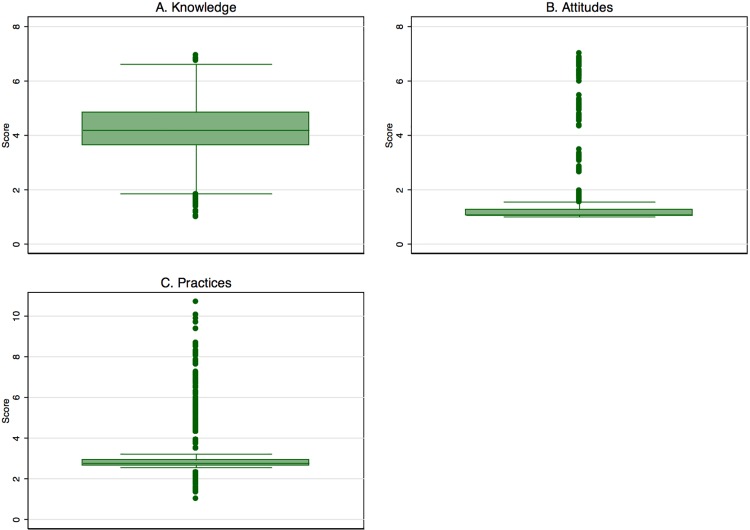
Distribution of the scores of knowledge, attitudes and practices. **KAP profiles.** Box plots of the scores of knowledge, attitudes and practices A. Knowledge profile scores are evenly distributed around the median with few outliers, predominantly below 1.8. B. Attitude scores are skewed to the right and values rages between 1 and 7. C. Practices is also skewed to the right with a range between 1 and 11.

We used MCA to calculate a score for each KAP domain; the first dimension explained 56.13%, 79.66%, and 83.16% of the variances, respectively. The knowledge score had inertia of 0.01 (66 variables), the average score was 4.24 (std. dev. = 1), and the maximum value was 6.96. The attitude score had inertia of 0.122 (17 variables), with a mean score of 1.40 (std. dev. = 1), and the maximum value was 7.02. The practices score had inertia of 0.05, an average of 3.18 (std. dev. = 1.1), and a maximum value of 10.68 (Fig 1).

As a result of the hierarchical cluster analysis, we determined five profiles in the knowledge domain according to the score generated using MCA. Profile 1 was characterized by participants not having heard about the disease and no reported knowledge about any feature of the means of transmission, clinical presentation, characteristics of *Aedes aegypti*, or prevention measures. Profile 2 entailed individuals who despite having heard about dengue and its means of transmission did not know about preventive measures or any other aspect of dengue or the vector. Profiles 3 and 4 included individuals who had knowledge about oviposition places (any stagnant water) and means of transmission. Additionally, individuals assigned to profile 4 named more constitutional symptoms, while those in profile 3 named more hemorrhagic symptoms (such as petechiae, epistaxis, etc.). Profile 5 was characterized by a high knowledge about the means of transmission and recognition of the white-striped legs of the vector (Table 2A).

**Table 2:** Profiles of Knowledge and practices.

A. Knowledge						
	Heard about dengue	Transmission	Vector Characteristics	Symptoms	Oviposition	Prevention
Profile 1	YES	YES	YES	YES	YES	YES
Profile 2	YES	YES	YES	YES	YES	YES
Profile 3	YES	YES	YES	NO	NO	NO
Profile 4	YES	YES	NO	NO	NO	NO
Profile 5	NO	NO	NO	NO	NO	NO
B. Practices						
	Coverage of water containers	Water treatment	Education to other members of the household	Emptying frequency of water containers > 7 days		
Profile 1	NO	NO	NO	NO		
Profile 2	NO	NO	NO	NO		
Profile 3	NO	NO	NO	YES		
Profile 4	YES	YES	YES	YES		
Profile 5	YES	YES	YES	YES		
Profile 6	YES	YES	YES	YES		
Profile 7	YES	YES	YES	YES		

Attitude analysis generated nine profiles that did not show specific patterns per profile in the components of attitudes but could be grouped into two types: the individuals who thought that dengue is important to the community and to them, and the ones who did not. The remaining variables such as considering dengue as a serious disease and that dengue is an issue for the community and for them were evenly distributed across profiles. However, the first group accounted for 95% of the individuals, revealing that there was not enough variance between the groups. Moreover, no meaningful pattern was identified when categorizing into quartiles. For this reason, this domain was excluded from the subsequent phases of the analysis.

Practices scores resulted in seven profiles. Profiles 1 and 2 were characterized by poor prevention practices against vectors, such as no coverage of water containers or water treatment, no education to other members of the household, and a low frequency of emptying water from containers more than seven days, regardless of its capacity. Persons who did not cover or add chemical substances to water containers, but who emptied water containers, were part of profile 3, and the best practices corresponded to profiles 4, 5, 6, and 7 (Table 2). The distribution of the profiles followed a descendant order, whereby the smallest score was in profile 1 and the highest in profile 7; for this reason, practices scores were treated as ordinal variables.

### Sociodemographic factors associated to KAP

#### Knowledge

The knowledge score was grouped in tertiles as follows: 6.80th, 50th, and 87.24^th^, from low to high according to the scores of the profiles from the cluster analysis. The first tertile (low knowledge) grouped values from 1 to 2.79 and corresponded to the group of individuals who had not heard about dengue or the ones who reported poor knowledge about the disease, the vector, or preventive measures (profiles 4 and 5). The 50th tertile (medium knowledge) was composed of persons who knew about the means of transmission, oviposition places, and symptoms of the disease (profile 1 and 2), while the 87.24th tertile (high knowledge) grouped scores from 5.50 to 6.96 and included individuals who knew about all the features mentioned before and who could identify the color of *Aedes aegypti*.

Our estimation showed a positive relation between age and the knowledge score. This effect was heterogeneous by levels of knowledge. For individuals categorized with low knowledge, the marginal effect of age was 0.06 (IC: [0.03; 0.09]), and for individuals in the medium- and high-knowledge categories, the effects were 0.03 (IC: [0.02, 0.04]) and 0.02 (IC: [0.01; 0.03]), respectively. Years of education also had a positive relation with this score for the low- and medium-level knowledge group, with marginal effects of 0.14 (IC: [0.06; 0.22]) and 0.04 (IC: [0.01; 0.07]). However, there was no significant association with the high-level group.

Among household members, the number of persons dedicated to housewifery decreased the score by 0.11 (IC: [-0.15; -0.08]) in the medium-level knowledge group and by 0.08 (IC: [-0.15; -0.01]) in the high-level knowledge group, in comparison to any other occupation reported. Additionally, for each household member with a history of dengue, the score rises by 0.21 (IC: [0.12, 0.31]) in the low-level knowledge group and 0.07 (IC: [0.01; 0.12]) in the medium-level group and did not show an effect in the group with the highest knowledge. Variables such as sex, socioeconomic strata, the number of women in the household, and migration were not statistically significant, as seen in Table 3.

**Table 3:** Factors associated to low medium and high levels of knowledge, Armenia and Arauca.

	Low	Medium	High
VARIABLES	*6*.*8*^*th*^ *quantile*	*50*^*th*^ *quantile*	*87*.*24*^*th*^ *quantile*
Years of education	0.14***	0.04**	0
	(0.06–0.22)	(0.01–0.07)	(-0.03–0.03)
Age	0.06***	0.03***	0.02***
	(0.03–0.09)	(0.02–0.04)	(0.01–0.03)
Sex	0.07	-0.02	-0.05
	(-0.13–0.28)	(-0.10–0.07)	(-0.15–0.04)
Income: Less than 1 MS	0.34**	0.38***	0.51***
	(0.02–0.67)	(0.28–0.48)	(0.40–0.62)
Income: 1–2 MS	0.47***	0.39***	0.51***
	(0.24–0.69)	(0.28–0.49)	(0.41–0.62)
Income 2–3 MS	0.17	0.15**	0.23***
	(-0.14–0.47)	(0.02–0.28)	(0.14–0.33)
income: 3–4 MS	0.47***	0.14**	0.28***
	(0.14–0.80)	(0.01–0.28)	(0.11–0.45)
income: 4–5 MS	0.66**	0.34**	0.2
	(0.14–1.17)	(0.03–0.64)	(-0.13–0.52)
income: more than 5 MS	0.55**	0.21**	0.24*
	(0.09–1.01)	(0.02–0.41)	(-0.04–0.52)
Socioeconomic Stratum	-0.03	-0.07**	0.02
	(-0.14–0.08)	(-0.13–-0.00)	(-0.05–0.08)
Number of workers per household	-0.05	-0.02	0.02
	(-0.13–0.04)	(-0.06–0.02)	(-0.04–0.08)
Number of unemployed per household	0.17*	0.04	0
	(-0.01–0.34)	(-0.05–0.13)	(-0.10–0.10)
Number of habitants dedicated to housework per household	-0.08	-0.11***	-0.08**
	(-0.19–0.04)	(-0.15–-0.08)	(-0.15–-0.01)
Number of women per household	-0.02	0.02	0.03
	(-0.09–0.06)	(-0.02–0.06)	(-0.01–0.07)
Number of dengue cases per household	0.21***	0.07**	0.02
	(0.12–0.31)	(0.01–0.12)	(-0.03–0.07)
Person who decide about: personal healthcare			
*Men*	-0.08	-0.49	-0.38
* *	(-1.26–1.10)	(-1.60–0.62)	(-0.86–0.10)
*Women*	-0.23	-0.57	-0.52**
* *	(-1.44–0.98)	(-1.67–0.53)	(-1.02–-0.01)
*Both*	-0.34	-0.52	-0.51*
	(-1.46–0.79)	(-1.59–0.56)	(-1.03–0.02)
Person who decide about: family healthcare			
*Men*	0.80*	0.37*	0.41***
* *	(-0.03–1.62)	(-0.00–0.75)	(0.19–0.63)
*Women*	0.75	0.37**	0.41***
* *	(-0.15–1.64)	(0.07–0.67)	(0.26–0.56)
*Both*	0.87**	0.56***	0.49***
	(0.10–1.63)	(0.27–0.86)	(0.32–0.67)
Person who decide about: Household chores			
*Men*	-2.01	-0.27	0.45
* *	(-4.69–0.66)	(-1.78–1.25)	(-0.85–1.75)
*Women*	-1.95	-0.35	0.36
* *	(-4.53–0.62)	(-1.81–1.11)	(-0.90–1.62)
*Both*	-1.99	-0.3	0.48
	(-4.56–0.58)	(-1.79–1.19)	(-0.80–1.76)
High expenses in the household			
*Men*	0.14	0.24	0.06
* *	(-1.64–1.92)	(-1.06–1.55)	(-1.31–1.42)
*Women*	0.36	0.3	0.17
* *	(-1.47–2.19)	(-0.99–1.58)	(-1.20–1.54)
*Both*	0.48	0.28	0.04
	(-1.27–2.24)	(-1.00–1.56)	(-1.29–1.36)
Daily expenses in the household			
*Men*	1.06	1.14	0.1
* *	(-0.98–3.11)	(-0.74–3.01)	(-1.65–1.85)
*Women*	1.05	1.36	0.28
* *	(-1.01–3.11)	(-0.52–3.24)	(-1.44–2.00)
*Both*	1.07	1.21	0.25
	(-0.85–2.99)	(-0.69–3.11)	(-1.49–1.99)
Constant	0.56	2.31***	3.98***
	(-1.12–2.24)	(1.07–3.55)	(2.91–5.05)
Observations	3725	3725	3725

Confidence interval in parentheses

*** p<0.01

** p<0.05

* p<0.

This regression estimates where controlled by effects due to number of inhabitants per household, numbers of years living in the neighborhood or dwelling, and, non-linear effects of years of education and age.

The effect of joint (male and female) versus individual decision making regarding family care was higher across all knowledge groups (Table 3). Decisions about the health of all members of the household made by male, female, or collectively showed an increase of the score in all knowledge groups by at least 0.50 (IC: [0.32; 0.67]). Decisions about major expenses did not seem to have an impact on knowledge score.

#### Practices

The score was divided into two quantiles: the 25th and 75th percentiles. The first grouped scored between 1 and 2.67 and the second between 2.09 and 10.68. In the multivariable analysis, none of the socioeconomic factors, such as age, sex, years of education, and strata, were determinants of the practices scores. Gender decision making did not show a correlation either.

## Discussion

To our knowledge, this is the first time that MCA has been applied to analyze knowledge, attitudes, and practices regarding dengue to generate an ordinal score from a data set with several categorical variables. The impact of years of education and history of dengue among households increased dengue knowledge only among low- and medium-level knowledge profiles. The effect of more than one person reporting housekeeping in the same household as their principal occupation had a negative effect on the middle- and high-level knowledge scores. Furthermore, decision making about family health care shared by men and women increased the score of knowledge at any level. Finally, practices scores were not related to any of the measured sociodemographic or gender decision-making variables.

Age and education have also been identified as the only sociodemographic variables associated with more knowledge about dengue by other studies in Thailand [50,51], Malaysia [52], Cuba (only age) [20], Indonesia [13], and Jamaica (only education) [53]. Two studies, one in Laos and one in Malaysia, reported no statistical association between age or education with the ability to name more than one symptom of dengue [17,54]. In our study, we found that education levels have a positive relationship with the improvement of knowledge up to a certain point, and it does not show an effect on the higher score level.

The described relationship might be caused by the decrease in variance of the households with the highest knowledge score, which does not allow us to detect differences between education levels. However, this finding can also be another manifestation of the previously documented “base education hypothesis,” in which the effect of education is not linear—beyond 12 years of formal education attained (corresponding to a high school level), it does not seem to affect other outcomes in health [43]. In this case, higher levels of outcome cannot be achieved solely by increasing education, and it is suggestive that the underlying mechanisms for having detailed knowledge of dengue, such as the color of the mosquito’s legs, are different from those for middle and low levels of knowledge. Examples of such mechanisms, which are in accord with other results of this study, are the levels of empowerment of the family and their access to information, as hypothesized by Cutler and Oreopolus in their study about mortality [55,56].

Studies in other settings have found an association between socioeconomic status (SES) and knowledge; however, there are several ways to measure SES and knowledge. While Castro et al. used a household asset score [20] in Cuba, and Itrat used monthly income [14] in Pakistan, in this study, we used a socioeconomic stratification system utilized by the government that entails characteristics of the neighborhood and income among others [40]. The lack of a significant association in this study could be due to accounting for the confounding effect of clustering in the relationship between SES and the degree of knowledge regarding dengue. SES, as measured in the study, is clustered in neighborhoods, and other studies in the area have shown that knowledge of dengue is also clustered by neighborhoods [57].

In our study, having more than one individual within a household reporting “housekeeping” as his or her main activity during the 10 days prior to the survey indicated a negative effect on the knowledge score. This suggests that there are higher levels of informal occupations that could not be captured in the questionnaire [58]. Informality is often associated with households facing poorer economic conditions, which creates difficulty in collecting accurate data. The observed relationship with the knowledge score is identifying a different component of SES that is not captured by income or education. Further exploration of the conditions of the population that reported more than one housekeeper should be explored.

The findings of this study highlight the role of joint decision making between men and women in the family’s health care as a factor that contributes to the knowledge of dengue and its transmission. Past studies have suggested the need to approach the role of gender in the distribution of household chores and its relationship with dengue [57,59]. For this study, gender roles were approached from a micro sociological perspective, in which the dynamics on a small scale (families and couples) can be observed through decision-making processes [60].

Our results evidenced that shared decision-making processes between men and women play a significant role in the acquisition of knowledge about dengue; this finding has also been observed in contraception [61–63] and malaria [64] studies, in which such an effect is explained by a higher capacity of communication and negotiation. It seems that these two elements serve as a mechanism for consolidation, providing a better understanding of dengue. These findings and its congruence with other health outcomes pave the road to further exploration of the mechanisms in which joint decision making improves knowledge and empowerment within the household across the health spectrum [63].

Although many studies have conducted KAP surveys, few studies have addressed the question of the associated factors to preventive practices for dengue, and most of them have described these practices or their association with immature forms of the vector [54,65,66]. Despite this, our findings of a lack of significant association between practices score and sociodemographic characteristics are also found in other studies (in Jamaica, Cuba, and Vietnam) in which the authors suggest that cultural factors could lead to certain practices [20,53,67,68]. This hypothesis has been addressed by other studies and is a growing interdisciplinary field [69].

The main recommendation of the World Health Organization (WHO) and the Pan American Health Organization (PAHO) is to control the immature forms of *Aedes aegypti* for the recent Zika and Chikungunya outbreaks in the Americas [70,71]. This study suggests the need for further assessment of the determinants of the practices of vector control that move beyond sociodemographic factors. Moreover, it provides an additional tool for tackling the routine questionnaires performed during vector control campaigns.

Since this is a cross-sectional analysis, one limitation is the impossibility of establishing the temporality of the relationships described. Moreover, even when considering fixed effects that allow controlling for the correlation between households of the same cluster, it is not possible to control for unmeasured confounding variables that vary over time, such as seasonal preventive interventions in some neighborhoods or unequal access to media that could confound the effect of sociodemographic characteristics on knowledge scores.

Even though selection bias is a possibility, we think there are mainly three reasons for not thinking this will affect our results. The first reason is mainly because we think that since we were assessing household behavior there was no better informant than the housewife itself, most of the times when asking other person in the house they would refer or even ask the housewife about some of the practices. The second reason is that 30% of the women reported working rather than housewifery. This indicates that recruitment time also allowed us to have information about women whose main activity was different from housewifery. Finally, when exploring other studies in which KAP about dengue was done 5 out 8 reported more or equal to 50% of its participants as housewives [18,72–75] and only 3 reported a proportion of less than 20% [51,52,54]. This makes us think that this might be a characteristic of the type of survey that we are doing rather than a bias.

In spite of the previous literature search and collection of most of the KAP questionnaires applied in the region for the development of the survey, comparability with other studies was a challenge. It would be helpful to generate a standardized and validated KAP questionnaire that allows comparisons between countries and across time. The use of factor analysis would facilitate the validation of such tools [37], however it is of crucial importance to reach a consensus regarding the definition of each of the domains and what each knowledge, attitude and practices construct means.

Additionally, it is necessary to establish a more suitable way to address attitudes, given that the heterogeneity of the responses in the context of a survey does not allow generalizations in the study population, our results provide evidence that KAP surveys have important measurement limitations. As discussed previously by Launiala [21] the measurement of attitudes via surveys is a sensitive topic, independent of the health issue of interest [76]. Measurement constrains such as respondent bias e.g. faulty recall and social desirability [77] question the validity of this KAP surveys and raise concerns about the possibility of measuring attitudes through surveys. Qualitative approaches such as interviews and direct observation may be more adequate as they allow rapport and buffer cultural barriers between the researchers and the respondents.

Our results show that in the context of our study the attitudes domain cannot be summarized into one or two variables because of the heterogeneity and discordance in the data collected. Further research in this issue may provide evidence of these patterns in the context of other public health issues, which will vary given for example socio-cultural norms about the health outcome of interest. A mixed methods approach may be ideal as a methodological strategy to triangulate information about a culturally and socially sensitive topic [78].

MCA, besides from being a generalization of correspondence analysis (CA), can be an adequate data procedure to reduce and summarize a large number of categorical variables into one ordinal variable, where the weighting process is due to a maximization of the overall correlation structure. It is helpful to understand the factors that might contribute to different levels of KAP in the community beyond the traditional descriptive analysis. Additionally, it can also be used as a tool to identify ways to improve the questionnaire and the classification of individuals into categories. Finally, given the broad use of KAP surveys in many aspects of public health such as research, planning and evaluating interventions across different issues in health, MCA becomes a useful tool to analyze the vast amount of data collected with a KAP questionnaire by the creation of one index, optimizing interpretation and usefulness.

This study allowed us to identify multiple research opportunities, including the further use of this method to determine what levels of knowledge are associated with pupal indexes, to validate KAP surveys in different populations, to conduct further reliability studies, and to implement an abbreviated version of the method. Since the current evidence about the drivers of preventive practices against dengue is not conclusive, further exploration of such factors would help policy makers to understand and thus promote them in the population at risk.

In conclusion, MCA is a useful tool for the analysis of KAP surveys. In regard to dengue, age and education are the only socio demographic factors associated with lower or mid levels of knowledge, whereas collective decision-making processes in the household are positively related to high levels of knowledge. No sociodemographic factors were associated with practices.

## Supporting Information

S1 File KAP Questionnaire. (PDF)

## References

[1] Guzman MG, Halstead SB, Artsob H, Buchy P, Farrar J, Gubler DJ, et al Dengue: a continuing global threat. Nat Rev Microbiol [Internet]. 2010 12 [cited 2013 Aug 7];8(12 Suppl):S7–16. Available from: http://www.ncbi.nlm.nih.gov/pubmed/21079655 10.1038/nrmicro2460 21079655 PMC4333201

[2] World Health Organization. Fact sheet 117: Dengue and severe dengue [Internet]. Fact Sheets. World Health Organization; 2014 [cited 2014 Jul 10]. p. 2. Available from: http://www.who.int/mediacentre/factsheets/fs117/en/

[3] San Martín JL, Brathwaite O, Zambrano B, Solórzano JO, Bouckenooghe A, Dayan GH, et al The epidemiology of dengue in the americas over the last three decades: a worrisome reality. Am J Trop Med Hyg [Internet]. 2010 1 [cited 2013 Aug 9];82(1):128–35. Available from: http://www.pubmedcentral.nih.gov/articlerender.fcgi?artid=2803522&tool=pmcentrez&rendertype=abstract 10.4269/ajtmh.2010.09-0346 20065008 PMC2803522

[4] (OPS/OMS) OP de la SM de la S. Información y Análisis de Salud (HSD/HA): Situación de Salud en las Américas: Indicadores Básicos 2012 [Internet]. Washington D.C; 2012. Available from: http://www2.paho.org/saludenlasamericas/dmdocuments/ib-2012-spa.pdf

[5] Brathwaite Dick O, San Martín JL, Montoya RH, del Diego J, Zambrano B, Dayan GH. The history of dengue outbreaks in the Americas. Am J Trop Med Hyg [Internet]. 2012 10 [cited 2013 Aug 7];87(4):584–93. Available from: http://www.ncbi.nlm.nih.gov/pubmed/23042846 10.4269/ajtmh.2012.11-0770 23042846 PMC3516305

[6] World Health Organization. Handbook for integrated vector management [Internet]. 1st ed. World Health Organization, editor. Outlooks on Pest Management. Geneva: World Health Organization; 2012. 1–143 p. Available from: http://openurl.ingenta.com/content/xref?genre=article&issn=1743-1026&volume=24&issue=3&spage=142

[7] Bota R, Ahmed M, Jamali MS, Aziz A. Knowledge, attitude and perception regarding dengue fever among university students of interior Sindh. J Infect Public Health [Internet]. 2014 1 [cited 2015 Jul 30];7(3):218–23. Available from: http://www.sciencedirect.com/science/article/pii/S1876034114000112 10.1016/j.jiph.2013.11.004 24613408

[8] Phuong HL, De Vries PJ, Boonshuyar C, Binh TQ, Nam N V, Kager PA. Dengue risk factors and community participation in Binh Thuan Province, Vietnam, a household survey. Southeast Asian J Trop Med Public Health [Internet]. 2008 1 [cited 2014 Jan 23];39(1):79–89. Available from: http://www.ncbi.nlm.nih.gov/pubmed/18567446 18567446

[9] Saied KG, Al-Taiar A, Altaire A, Alqadsi A, Alariqi EF, Hassaan M. Knowledge, attitude and preventive practices regarding dengue fever in rural areas of Yemen. Int Health [Internet]. 2015;7(6):420–5. Available from: http://inthealth.oxfordjournals.org/cgi/doi/10.1093/inthealth/ihv021 10.1093/inthealth/ihv021 25858280

[10] Wong LP, AbuBakar S, Chinna K. Community Knowledge, Health Beliefs, Practices and Experiences Related to Dengue Fever and Its Association with IgG Seropositivity. PLoS Negl Trop Dis [Internet]. Public Library of Science; 2014 5 22;8(5):e2789 Available from: http://dx.doi.org/10.1371%2Fjournal.pntd.0002789 10.1371/journal.pntd.0002789 24853259 PMC4031145

[11] Winch PJ, Leontsini E, Rigau-Pérez JG, Ruiz-Pérez M, Clark GG, Gubler DJ. Community-based dengue prevention programs in Puerto Rico: impact on knowledge, behavior, and residential mosquito infestation. Am J Trop Med Hyg [Internet]. 2002 10 [cited 2014 Jan 27];67(4):363–70. Available from: http://www.ncbi.nlm.nih.gov/pubmed/12452490 12452490 10.4269/ajtmh.2002.67.363

[12] Hairi F, Ong C-HS, Suhaimi A, Tsung T-W, bin Anis Ahmad MA, Sundaraj C, et al A knowledge, attitude and practices (KAP) study on dengue among selected rural communities in the Kuala Kangsar district. Asia Pac J Public Health [Internet]. 2003 1 [cited 2014 Jan 23];15(1):37–43. Available from: http://www.ncbi.nlm.nih.gov/pubmed/14620496 14620496 10.1177/101053950301500107

[13] Porter KR, Beckett CG, Kosasih H, Tan RI, Alisjahbana B, Rudiman PIF, et al Epidemiology of dengue and dengue hemorrhagic fever in a cohort of adults living in Bandung, West Java, Indonesia. Am J Trop Med Hyg [Internet]. 2005 1 [cited 2015 Jun 26];72(1):60–6. Available from: http://www.ncbi.nlm.nih.gov/pubmed/15728868 15728868

[14] Itrat A, Khan A, Javaid S, Kamal M, Khan H, Javed S, et al Knowledge, awareness and practices regarding dengue fever among the adult population of dengue hit cosmopolitan. PLoS One [Internet]. 2008 1 [cited 2014 Jan 22];3(7):e2620 Available from: http://www.pubmedcentral.nih.gov/articlerender.fcgi?artid=2440812&tool=pmcentrez&rendertype=abstract 10.1371/journal.pone.0002620 18612437 PMC2440812

[15] Ibrahim NKR, Al-Bar A, Kordey M, Al-Fakeeh A. Knowledge, attitudes, and practices relating to Dengue fever among females in Jeddah high schools. J Infect Public Health [Internet]. 2009 1 [cited 2014 Jan 23];2(1):30–40. Available from: http://www.ncbi.nlm.nih.gov/pubmed/20701858 10.1016/j.jiph.2009.01.004 20701858

[16] Ashok Kumar V, Rajendran R, Manavalan R, Tewari SC, Arunachalam N, Ayanar K, et al Studies on community knowledge and behavior following a dengue epidemic in Chennai city, Tamil Nadu, India. Trop Biomed [Internet]. 2010 8 [cited 2014 Jan 23];27(2):330–6. Available from: http://www.ncbi.nlm.nih.gov/pubmed/20962733 20962733

[17] Al-Dubai SAR, Ganasegeran K, Mohanad Rahman A, Alshagga MA, Saif-Ali R. Factors affecting dengue fever knowledge, attitudes and practices among selected urban, semi-urban and rural communities in Malaysia. Southeast Asian J Trop Med Public Health [Internet]. 2013 1 [cited 2014 Jan 23];44(1):37–49. Available from: http://www.ncbi.nlm.nih.gov/pubmed/23682436 23682436

[18] Paz-Soldán VA, Morrison AC, Lopez JJC, Lenhart A, Scott TW, Elder JP, et al Dengue knowledge and preventive practices in Iquitos, Peru. Am J Trop Med Hyg. 2015;93(6):1330–7. 10.4269/ajtmh.15-0096 26503276 PMC4674254

[19] Elsinga J, Lizarazo EF, Vincenti MF, Schmidt M, Velasco-Salas ZI, Arias L, et al Health Seeking Behaviour and Treatment Intentions of Dengue and Fever: A Household Survey of Children and Adults in Venezuela. PLoS Negl Trop Dis. 2015;9(12):1–18. 10.1371/journal.pntd.0004237 PMC4666462 26624283

[20] Castro M, Sánchez L, Pérez D, Sebrango C, Shkedy Z, Van der Stuyft P. The Relationship between Economic Status, Knowledge on Dengue, Risk Perceptions and Practices. PLoS One [Internet]. Public Library of Science; 2013 12;8(12):1–6. Available from: 10.1371/journal.pone.0081875 10.1371/journal.pone.0081875 PMC3861357 24349145

[21] Launiala A. How much can a KAP survey tell us about people’s knowledge, attitudes and practices? Some observations from medical anthropology research on malaria in pregnancy in Malawi. Anthropol Matters. 2009;11(1).

[22] Krentel A, Fischer P, Manoempil P, Supali T, Servais G, Rückert P. Using knowledge, attitudes and practice (KAP) surveys on lymphatic filariasis to prepare a health promotion campaign for mass drug administration in Alor District, Indonesia. Trop Med Int Health [Internet]. 2006 11 [cited 2016 Jul 3];11(11):1731–40. Available from: http://www.ncbi.nlm.nih.gov/pubmed/17054754 17054754 10.1111/j.1365-3156.2006.01720.x

[23] Eversole MSA, Bammek J. A kap study on malaria in Zanzibar: implications for prevention and controlA study conducted for unicef Sub-Office Zanzibar. Eval Program Plann. 1998;21(4):409–13.

[24] Salonen JT, Nyyssönen K, Salonen R, Lakka HM, Kaikkonen J, Porkkala-Sarataho E, et al Antioxidant Supplementation in Atherosclerosis Prevention (ASAP) study: a randomized trial of the effect of vitamins E and C on 3-year progression of carotid atherosclerosis. J Intern Med [Internet]. 2000 11 [cited 2016 Jul 3];248(5):377–86. Available from: http://www.ncbi.nlm.nih.gov/pubmed/11123502 11123502 10.1046/j.1365-2796.2000.00752.x

[25] Sarraf-Zadegan N, Sadri G, Malek Afzali H, Baghaei M, Mohammadi Fard N, Shahrokhi S, et al Isfahan Healthy Heart Programme: a comprehensive integrated community-based programme for cardiovascular disease prevention and control. Design, methods and initial experience. Acta Cardiol [Internet]. 2003 8 [cited 2016 Jul 3];58(4):309–20. Available from: http://www.ncbi.nlm.nih.gov/pubmed/12948036 10.2143/AC.58.4.2005288 12948036

[26] Richards FO, Klein RE, de León O, Mendizábal-Cabrera R, Morales AL, Cama V, et al A Knowledge, Attitudes and Practices Survey Conducted Three Years after Halting Ivermectin Mass Treatment for Onchocerciasis in Guatemala. PLoS Negl Trop Dis [Internet]. 2016 6 [cited 2016 Jul 4];10(6):e0004777 Available from: http://www.ncbi.nlm.nih.gov/pubmed/27341104 10.1371/journal.pntd.0004777 27341104 PMC4920414

[27] Tabash MI, Hussein RA, Mahmoud AH, El-Borgy MD, Abu-Hamad BA. Impact of an intervention programme on knowledge, attitude and practice of healthcare staff regarding pharmaceutical waste management, Gaza, Palestine. Public Health [Internet]. Elsevier; 2016 6 8 [cited 2016 Jul 4]; Available from: http://www.publichealthjrnl.com/article/S0033350616300361/fulltext 10.1016/j.puhe.2016.04.001 27289258

[28] Berman PA. Rethinking health care systems: Private health care provision in India. World Dev [Internet]. Pergamon; 1998 8 [cited 2016 Jul 4];26(8):1463–79. Available from: http://linkinghub.elsevier.com/retrieve/pii/S0305750X9800059X

[29] Filmer D, Pritchett LH. Estimating Wealth Effects without Expenditure Data—Or Tears: An Application to Educational Enrollments in States of India. Demography [Internet]. Wold Bank; 2001 2;38(1):115–32. Available from: http://link.springer.com/journal/volumesAndIssues/13524 11227840 10.1353/dem.2001.0003

[30] Greenacre M, Blasius J. Multiple correspondence analysis and related methods. CRC Press; 2006.

[31] Booysen F, van der Berg S, Burger R, Maltitz M von, Rand G du. Using an Asset Index to Assess Trends in Poverty in Seven Sub-Saharan African Countries. World Dev [Internet]. 2008 6 [cited 2015 May 11];36(6):1113–30. Available from: http://www.sciencedirect.com/science/article/pii/S0305750X08000466

[32] Wabiri N, Taffa N. Socio-economic inequality and HIV in South Africa. BMC Public Health [Internet]. BMC Public Health; 2013;13(1):1037 Available from: http://www.ncbi.nlm.nih.gov/pubmed/24180366 24180366 10.1186/1471-2458-13-1037 PMC4228412

[33] Avila D, Keiser O, Egger M, Kouyos R, Böni J, Yerly S, et al Social Meets Molecular: Combining Phylogenetic and Latent Class Analyses to Understand HIV-1 Transmission in Switzerland. Am J Epidemiol [Internet]. 2014 6 15;179 (12): 1514–25. Available from: http://aje.oxfordjournals.org/content/179/12/1514.abstract 10.1093/aje/kwu076 24821749

[34] Batista Ferrer H, Audrey S, Trotter C, Hickman M. An appraisal of theoretical approaches to examining behaviours in relation to Human Papillomavirus (HPV) vaccination of young women. Prev Med (Baltim) [Internet]. 2015 12 [cited 2015 Dec 18];81:122–31. Available from: http://www.ncbi.nlm.nih.gov/pubmed/26314783 10.1016/j.ypmed.2015.08.004 PMC4728193 26314783

[35] Batista-Foguet JM, Mendoza R, Perez-Perdigon M, Rius R. Life-Styles of Spanish School-Age Children: Their Evolution over Time. Use of Multiple Correspondence Analysis to Determine Overall Trends over Time in a Sequential, Cross-Sectional Study. New Aprroaches in Applied Statistics. 2000;173–210.

[36] Suzuki K, Mutinelli LE. Knowledge and practices about hantavirus pulmonary syndrome in a cluster of Japanese communities in Argentina. Rev Panam Salud Publica [Internet]. 2009;25(2):128–33. Available from: http://www.ncbi.nlm.nih.gov/pubmed/19531307 19531307 10.1590/s1020-49892009000200005

[37] DeVellis RF. Scale development: Theory and applications. Sage publications; 2016.

[38] Kohn JL. What is Health? A Multiple Correspondence Health Index. East Econ J [Internet]. Nature Publishing Group; 2012 Jun 6 [cited 2014 Mar 5];38(2):223–50. Available from: http://www.palgrave-journals.com/doifinder/10.1057/eej.2011.5

[39] Quintero J, García-Betancourt T, Cortés S, García D, Alcalá L, González-Uribe C, et al Effectiveness and feasibility of long-lasting insecticide-treated curtains and water container covers for dengue vector control in Colombia: a cluster randomised trial. Trans R Soc Trop Med Hyg [Internet]. Oxford University Press; 2015 2 19;109(2):116–25. Available from: http://www.ncbi.nlm.nih.gov/pmc/articles/PMC4299530/ 10.1093/trstmh/tru208 25604762 PMC4299530

[40] Castaneda T, Fernández L. Targeting social spending to the poor with proxy-means testing: Colombia’s SISBEN system [Internet]. World Bank. Washington, DC. Processed. Washington, DC; 2005. Report No.: 529. Available from: http://documents.worldbank.org/curated/en/2005/06/6025315/targeting-social-spending-poor-proxy-means-testing-colombias-sisben-system

[41] Esbjørn A, Fjalland-Perez EL. Colombia: Social stratification by law [Internet]. International Federation for Housing and Planning. 2012 [cited 2016 Jan 1]. p. 1–2. Available from: http://www.ifhp.org/ifhp-blog/colombia-social-stratification-law

[42] ICF International. Demographic and Health Surveys Methodology—Questionnaires: Household, Woman’s, and Man’s. Measure DHS Phase III [Internet]. Calverton, Maryland USA; 2011. p. 1–7. Available from: http://www.measuredhs.com/publications/publication-DHSQ6-DHS-Questionnaires-and-Manuals.cfm

[43] Park JE. Textbook of preventive and social medicine.(A treatise on community health.). Textb Prev Soc Med treatise community Heal. Jabalpur: Banarsidas Bhanot; 1970;

[44] Raina S. Assessment of knowledge, attitude, and practice in health care delivery. N Am J Med Sci [Internet]. 2013 3 [cited 2014 Mar 26];5(3):249–50. Available from: http://www.pubmedcentral.nih.gov/articlerender.fcgi?artid=3632036&tool=pmcentrez&rendertype=abstract 10.4103/1947-2714.109226 23626968 PMC3632036

[45] Rounx B Le, Rouanet H. Geometric Data Analysis, from Correspondace to Structured Data Analysis. 2004.

[46] Kaufman L, Rousseeuw PJ. Finding Groups in Data: An Introduction to Cluster Analysis New York: Wiley; 1990.

[47] Jain AK, Dubes RC. Algorithms of Clustering Data Englewood Cliffs, NJ: Prentice Hall; 1988.

[48] Duda RO, Hart PE, Stork DG. Pattern Classification 2nd ed. New York: Wiley; 2001.

[49] StataCorp LP. STATA 12. College Station; 2012.

[50] Koenraadt CJM, Tuiten W, Sithiprasasna R, Kijchalao U, Jones JW, Scott TW. Dengue knowledge and practices and their impact on Aedes aegypti populations in Kamphaeng Phet, Thailand. Am J Trop Med Hyg [Internet]. 2006 4 [cited 2014 Jan 23];74(4):692–700. Available from: http://www.ncbi.nlm.nih.gov/pubmed/16607007 16607007

[51] Van Benthem BHB, Khantikul N, Panart K, Kessels PJ, Somboon P, Oskam L. Knowledge and use of prevention measures related to dengue in northern Thailand. Trop Med Int Health [Internet]. 2002 11 [cited 2014 Jan 27];7(11):993–1000. Available from: http://www.ncbi.nlm.nih.gov/pubmed/12390606 12390606 10.1046/j.1365-3156.2002.00950.x

[52] Naing C, Ren WY, Man CY, Fern KP, Qiqi C, Ning CN, et al Awareness of dengue and practice of dengue control among the semi-urban community: a cross sectional survey. J Community Health [Internet]. 2011 12 [cited 2014 Jan 23];36(6):1044–9. Available from: http://www.ncbi.nlm.nih.gov/pubmed/21528416 10.1007/s10900-011-9407-1 21528416

[53] Shuaib F, Todd D, Campbell-Stennett D, Ehiri J, Jolly PE. Knowledge, attitudes and practices regarding dengue infection in Westmoreland, Jamaica. West Indian Med J [Internet]. 2010 3 [cited 2014 Jan 23];59(2):139–46. Available from: http://www.pubmedcentral.nih.gov/articlerender.fcgi?artid=2996104&tool=pmcentrez&rendertype=abstract 21132094 PMC2996104

[54] Mayxay M, Cui W, Thammavong S, Khensakhou K, Vongxay V, Inthasoum L, et al Dengue in peri-urban Pak-Ngum district, Vientiane capital of Laos: a community survey on knowledge, attitudes and practices. BMC Public Health [Internet]. 2013 1 [cited 2014 Jan 23];13:434 Available from: http://www.pubmedcentral.nih.gov/articlerender.fcgi?artid=3645963&tool=pmcentrez&rendertype=abstract 10.1186/1471-2458-13-434 23641953 PMC3645963

[55] Cutler DM, Lleras-Muney A. Education and health: evaluating theories and evidence In: House J., Schoeni R., Kaplan G., Pollack H, editors. The Health Effects of Social and Economic Policy 2007 [Internet]. New York: Russell Sage Foundation; 2007 Available from: http://www.nber.org/papers/w12352.pdf

[56] Oreopoulos P, Salvanes KG. Priceless: The Nonpecuniary Benefits of Schooling. J Econ Perspect [Internet]. 2011;25(1):159–84. Available from: http://www.aeaweb.org/articles.php?doi=10.1257/jep.25.1.159

[57] Padmanabha H, Soto E, Mosquera M, Lord CC, Lounibos LP. Ecological links between water storage behaviors and Aedes aegypti production: implications for dengue vector control in variable climates. Ecohealth [Internet]. 2010 8 [cited 2014 Jan 22];7(1):78–90. Available from: http://www.ncbi.nlm.nih.gov/pubmed/20358255 10.1007/s10393-010-0301-6 20358255

[58] Hussmanns R. Measuring the informal economy: From employment in the informal sector to informal employment. Integr Work Pap. 2004;53.

[59] Pacheco-Coral A del P, Quiñones-Pinzón ML, Serrato-Pomar IM, Rivas-Muñoz FA. [Evaluating an Information, Education and Communication (IEC) strategy which was adopted for Aedes aegypti control in La Dorada, Colombia]. Rev Salud Publica (Bogota) [Internet]. 2010 6 [cited 2014 Jan 23];12(3):380–90. Available from: http://www.ncbi.nlm.nih.gov/pubmed/21311826 21311826 10.1590/s0124-00642010000300004

[60] Lindsey LL. Gender roles: A sociological perspective. Routledge; 2015.

[61] DeRose LF, Ezeh AC. Decision-making patterns and contraceptive use: evidence from Uganda. Popul Res Policy Rev. Springer; 2010;29(3):423–39.

[62] Speizer IS, Whittle L, Carter M. Gender Relations and Reproductive Decision Making in Honduras. Int Fam Plan Perspect [Internet]. Guttmacher Institute; 2005;31(3):131–9. Available from: http://www.jstor.org/stable/3649517 16263530 10.1363/3113105

[63] Upadhyay UD, Gipson JD, Withers M, Lewis S, Ciaraldi EJ, Fraser A, et al Women’s empowerment and fertility: a review of the literature. Soc Sci Med [Internet]. 2014 8 [cited 2015 Dec 28];115:111–20. Available from: http://www.sciencedirect.com/science/article/pii/S0277953614003736 10.1016/j.socscimed.2014.06.014 24955875 PMC4096045

[64] Tolhurst R, Nyonator FK. Looking within the household: gender roles and responses to malaria in Ghana. Trans R Soc Trop Med Hyg [Internet]. 2006 4 1;100 (4): 321–6. Available from: http://trstmh.oxfordjournals.org/content/100/4/321.abstract 16214194 10.1016/j.trstmh.2005.05.004

[65] Koyadun S, Butraporn P, Kittayapong P. Ecologic and sociodemographic risk determinants for dengue transmission in urban areas in Thailand. Interdiscip Perspect Infect Dis [Internet]. 2012 1 [cited 2015 Jun 24];2012:907494 Available from: http://www.pubmedcentral.nih.gov/articlerender.fcgi?artid=3463950&tool=pmcentrez&rendertype=abstract 10.1155/2012/907494 23056042 PMC3463950

[66] Syed M, Saleem T, Syeda U-R, Habib M, Zahid R, Bashir A, et al Knowledge, attitudes and practices regarding dengue fever among adults of high and low socioeconomic groups. J Pak Med Assoc [Internet]. 2010 3 [cited 2014 Jan 23];60(3):243–7. Available from: http://www.ncbi.nlm.nih.gov/pubmed/20225792 20225792

[67] Tsuzuki A, Huynh T, Tsunoda T, Luu L, Kawada H, Takagi M. Effect of existing practices on reducing Aedes aegypti pre-adults in key breeding containers in Ho Chi Minh City, Vietnam. Am J Trop Med Hyg [Internet]. 2009 5 [cited 2014 Jan 23];80(5):752–7. Available from: http://www.ncbi.nlm.nih.gov/pubmed/19407119 19407119

[68] Sanchez L, Perez D, Pérez T, Sosa T, Cruz G, Kouri G, et al Intersectoral coordination in Aedes aegypti control. A pilot project in Havana City, Cuba. Trop Med Int Health [Internet]. 2005 1;10(1):82–91. Available from: http://www.ncbi.nlm.nih.gov/pubmed/15655017 15655017 10.1111/j.1365-3156.2004.01347.x

[69] Arenas-Monreal L, Piña-Pozas M, Gómez-Dantés H. [Challenges and inputs of the gender perspective to the study of vector borne diseases]. Salud Publica Mex [Internet]. 1 [cited 2015 4 10];57(1):66–75. Available from: http://www.ncbi.nlm.nih.gov/pubmed/25629281 25629281

[70] World Health Organization. Fact Sheet: Zika virus [Internet]. Fact Sheets. World Health Organization; 2016 [cited 2016 Feb 24]. Available from: http://www.who.int/mediacentre/factsheets/zika/en/

[71] World Health Organization. Fact Sheet No. 327 Chikungunya [Internet]. Fact Sheets. World Health Organization; 2015 [cited 2016 Feb 24]. Available from: http://www.who.int/mediacentre/factsheets/fs327/en/

[72] Wong LP, AbuBakar S. Health Beliefs and Practices Related to Dengue Fever: A Focus Group Study. PLoS Negl Trop Dis [Internet]. Public Library of Science; 2013 7 11;7(7):e2310 Available from: http://dx.doi.org/10.1371%2Fjournal.pntd.0002310 10.1371/journal.pntd.0002310 23875045 PMC3708882

[73] Chandren JR, Wong LP, AbuBakar S. Practices of dengue fever prevention and the associated factors among the Orang Asli in Peninsular Malaysia. PLoS Negl Trop Dis. 2015;9(8):1–17. 10.1371/journal.pntd.0003954 PMC4534093 26267905

[74] Nalongsack S, Yoshida Y, Morita S, Sosouphanh K, Sakamoto J. Knowledge, attitude and practice regarding dengue among people in Pakse, Laos. Nagoya J Med Sci [Internet]. 2009 2 [cited 2014 Jan 23];71(1–2):29–37. Available from: http://www.ncbi.nlm.nih.gov/pubmed/19358473 19358473 PMC11166389

[75] Quintero J, Carrasquilla G, Suárez R, González C, Olano VA. An ecosystemic approach to evaluating ecological, socioeconomic and group dynamics affecting the prevalence of Aedes aegypti in two Colombian towns. Cad Saude Publica [Internet]. 2009 1 [cited 2014 Jan 23];25 Suppl 1:S93–103. Available from: http://www.ncbi.nlm.nih.gov/pubmed/19287871 19287871 10.1590/s0102-311x2009001300009

[76] Bacchus LJ, Buller AM, Ferrari G, Brzank P, Feder G. ‘It’s Always Good to Ask’: A Mixed Methods Study on the Perceived Role of Sexual Health Practitioners Asking Gay and Bisexual Men About Experiences of Domestic Violence and Abuse. J Mix Methods Res [Internet]. 2016 6 8; Available from: http://mmr.sagepub.com/content/early/2016/06/07/1558689816651808.abstract

[77] Gregson S, Zhuwau T, Ndlovu J, Nyamukapa CA. Methods to reduce social desirability bias in sex surveys in low-development settings: experience in Zimbabwe. Sex Transm Dis [Internet]. 2002 10 [cited 2016 Jul 4];29(10):568–75. Available from: http://www.ncbi.nlm.nih.gov/pubmed/12370523 12370523 10.1097/00007435-200210000-00002

[78] Hesse-Biber S. Doing Interdisciplinary Mixed Methods Health Care Research: Working the Boundaries, Tensions, and Synergistic Potential of Team-Based Research. Qual Health Res [Internet]. 2016 4 [cited 2016 Jul 4];26(5):649–58. Available from: http://www.ncbi.nlm.nih.gov/pubmed/26984708 10.1177/1049732316634304 26984708

