# Supplementary material for: KAP Surveys and Dengue Control in Colombia: Disentangling the Effect of Sociodemographic Factors Using Multiple Correspondence Analysis
Source: PLoS Negl Trop Dis. 2016 Sep 28;10(9):e0005016. doi: 10.1371/journal.pntd.0005016 (PMC5040257; doi:10.1371/journal.pntd.0005016)
Supplement: S1 File — (PDF) [file pntd.0005016.s001.pdf]

Supporting file 1. KAP Questionnaire

ID | | | | | | | |

Versión 10 - 28 de Noviembre de 2012

INVESTIGACION ECO-BIO-SOCIAL SOBRE DENGUE EN 2 MUNICIPIOS DE COLOMBIA

CUESTIONARIO DE HOGARES FASE 1

**I. INFORMACIÓN DE LA ENCUESTA Y GEORREFERENCIACIÓN**	
1. Nombre de quien realiza la encuesta:	
2. Fecha de la entrevista	dd ☐ mm ☐ aaaa ☐
3. Municipio	
4. Cluster	
6. Longitud	
7. Latitud	
5. Dirección del hogar	
8. Barrio	
9. Respuesta inicial: *P=Presente; A = Ausente; V.D = Vivienda no habitada*	

**II. INFORMACIÓN DEL ENCUESTADO**		
1. Cuales son sus nombres y apellidos?		
2. Telefono Fijo	No tiene telefono ☐	
3.. Celular	No tiene o no proporciona # ☐	
4. ¿En qué departamento y municipio nació?<br>Municipio<br>Departamento		
5. ¿En que més día y año nació usted?<br>1 = Día ☐ 2 = Mes ☐ 3 = Año ☐		
6. ¿Cuantos años cumplidos tiene?<br>VERIFIQUE QUE COINCIDA LA EDAD CON LA FECHA DE NACIMIENTO	☐	
7. Sexo *(1 = Masculino; 2 = Femenino)*	☐	
8. De acuerdo con su cultura, costumbres o rasgos físicos, usted es o se reconoce como:<br>*1 = Negro(a), afrodescendiente; 2 = Raizal del archipiélago; 3 = Palenquero; 4 = Gitano(a), Rom (Li); 5 = Indígena; 6 = Mestizo u otro diferente; 99=No sabe*	☐	
9. ¿Asiste actualmente a algún establecimiento educativo? *(1 = Sí; 2 = No)*	☐	
10. ¿Cuál fue el último año escolar que aprobó?<br>MARQUE CADA UNA DE LAS OPCIONES QUE APLIQUEN<br>Preescolar 1 ☐ 2 ☐ 3 ☐<br>Básica primaria 1 ☐ 2 ☐ 3 ☐ 4 ☐ 5 ☐<br>Básica secundaria 6 ☐ 7 ☐ 8 ☐ 9 ☐ 10 ☐ 11 ☐ 12 ☐<br>Técnico o tecnológico 1 ☐ 2 ☐ 3 ☐<br>Universitario 1 ☐ 2 ☐ 3 ☐ 4 ☐ 5 ☐ 6 ☐<br>Postgrado 1 ☐ 2 ☐ 3 ☐ 4 ☐ 5 ☐<br>Ninguno 0 ☐		
11. ¿En qué ocupó la mayor parte del tiempo la semana pasada de Lunes a Domingo?<br>*1 = Trabajando; 2 = No trabajó, pero tiene trabajo; 3 = Buscando trabajo; 4 = Estudiando; 5 = Oficios del hogar; 6 = Pensionado; 7 = Rentista; 8 = Incapacitado permanente; 9 = Otro:____________________; 10= No sabe*	☐☐	
12. Está afiliado o es beneficiario a Salud?<br>*(1 = Sí; 2 = No; 3 = No se sabe)*	☐	**SI "NO" PASE A 14**
13. ¿A cual entidad de salud está afiliado?<br>*1 = Entidad promotora de salud contributivo (EPS); 2 = Entidad promotora de salud subsidiado (EPSS); 3 = Especial (Fuerzas Militares/Policía Nacional, ECOPETROL, Magisterio; FONCOLPUERTOS); 9 = No está afiliado; 10= No sabe*	☐☐	
14. ¿En cuánto ubica el ingreso mensual de su familia? (NO LEA OPCIONES, ESPERE RESPUESTA)<br>(Suma del ingreso de todos los que aportan al mantenimiento de la familia)<br>*1 = <1 SMLV (0 a menos de $566.700); 2 = 1-2 SMLV (Entre $566.700 y $1.133.400); 3 = >2 y 3 SMLV (Entre $1.133.401 y$1.700.100); 4 = >3 y 4 SMLV (Entre $1.700.101 y $2.266.800); 5 = >4 - 5SMLV (Entre $2.266.801 y $2.833.500); 6 = >5 SMLV (Más de $2.833.501); 7 = No sabe*	☐	

15. ¿Usted vive solo? (1 = Sí; 2 = No)		**SI "SI" PASE A 17**
16. ¿Quien en su hogar tiene la última palabra en las siguientes deciciones?<br>*ESPERE RESPUESTA (1 = Un hombre; 2 = Una mujer; 3 = Ambos; 4 = Nadie; 5 = No Sabe)*		
1. El cuidado de su salud?		
2. El cuidado de la salud de los otros miembros del hogar?		
3. Hacer compras grandes del hogar?		
4. Hacer compras para necesidades diarias del hogar?		
5. El cuidado de la vivienda		
**III. MIGRACIÓN Y PERMANENCIA**		
17. ¿Durante cuánto tiempo ha vivido en este barrio?<br>Encuestador: No olvide incluír las unidades en su respuesta, por ejemplo años, meses, semanas		
18. ¿Por cuánto tiempo ha vivido en esta casa?<br>Encuestador: No olvide incluír las unidades en su respuesta, por ejemplo años, meses, semanas		
19. ¿Dónde vivía usted antes de venir a vivir a esta casa? (NO LEA OPCIONES, ESPERE RESPUESTA)<br>1 = Mismo municipio misma zona; 2 = Mismo municipio distinta zona (urbano /rural); 3 = Otro municipio de este departamento; 4 = Otro departamento; 5 = Otro país; 6 = No responde		
20. ¿Dónde vivía usted cuando ocurrió el terremoto de Armenia en 1999?<br><br>ENCUESTADOR: UNICAMENTE REALICE ESTA PREGUNTA SI ESTÁ APLICANDO ESTE CUESTIONARIO EN LA CIUDAD DE ARMENIA<br>NO LEA LAS OPCIONES, MARQUE LA OPCIÓN QUE EL ENCUESTADO RESPONDA ESPONTÁNEAMENTE<br>1 = Mismo barrio en Armenia; 2 = Otro barrio dentro de Armenia; 3 = Otro municipio de Quindío; 4 = Otro departamento; 5 = Otro país; 9 = No responde		

III. MIEMBROS DE LA VIVIENDA Y EL HOGAR (¿QUIÉNES VIVEN EN LA VIVIENDA?)				
21. ¿Cuantas personas viven en esta vivienda actualmente incluyendose a usted?				
22. ¿Cuantos hogares tiene la vivienda?				
23. ¿Cuántas personas viven en su hogar?				

Listado de miembros del hogar

	24. Nombre	25. Parentesco con el jefe del Hogar<br>(NO LEA OPCIONES, ESPERE RESPUESTA)<br>1 = Jefe del hogar<br>2 = Cónyuge/Compañer@<br>3 = Hij@<br>4 = Hij@ no biológic@<br>5 = Otro familiar<br>6 = No pariente	26. Cuantos años cumplidos tiene<br>(infante=0)	27. Sexo<br>1=M/2=F	28. ¿En qué ocupó la mayor parte del tiempo la semana pasada?<br>1 = Trabajando<br>2 = No trabajó, pero tiene trabajo<br>3 = Buscando trabajo<br>4 = Estudiando<br>5 = Oficios del hogar<br>6 = Pensionado<br>7 = Rentista<br>8 = Incapacitado permanente<br>9 = Otro:__________<br>10= No sabe	29. Un médico le ha diagnosticado Dengue alguna vez en la vida?<br>(1=Sí, 2=No, 3=No sabe)	30. ¿Cuántas veces ha sufrido dengue?	31. Cuándo fue la última vez que estuvo enfermo por dengue?<br>(**1=** Últimos 15 dias;<br>**2=** Hace un mes;<br>**3=** Hace más de 1 mes,<br>**4=** No sabe)	32. ¿En esa última vez, a dónde consultó por primera vez cuando comenzó la enfermedad?<br>(NO LEA OPCIONES, ESPERE RESPUESTA)<br>(**1=** Droguería o farmacia<br>**2=** Curandero o chaman<br>**3=** Vecina/o<br>**4 =** Familiar<br>**5 =** Centro de Salud<br>**6=** Hospital<br>**7=** Médico particular<br>**8 =** No consultó)
1									
2									
3									
4									
5									
6									
7									
8									
9									
10									
11									
12									
13									

IV. CARACTERÍSTICAS DE LA VIVIENDA Y SERVICIOS BÁSICOS

33. ¿Qué tipo de vivienda es esta?

1 = Casa; 2 = Apartamento; 3 = Cuarto(s) en inquilinato; 4 = Cuarto(s) en otro tipo de estructura; 5 = Otro tipo de vivienda (carpa, tienda, vagón, embarcación, refugio natural, puente, etc.)

34. ¿Cuál es el uso principal de su vivienda? (*1=Residencial; 2=Negocio; 3= Restaurante; 4=Mixto; 5=Otro)*

35. ¿Cuántos pisos tiene su vivienda? — **SI 1 PASE A 37**

36. Si hay más de uno, ¿En cuál vive usted?

37. OBSERVE Y ANOTE EL MATERIAL PREDOMINANTE DEL PISO DE LA VIVIENDA OCUPADA POR EL HOGAR

1 = Marmol, parqué, madera pulida y lacada; 2 = Alfombra, tapete de pared a pared; 3 = Baldosa, cerámica, vinilo, tableta, ladrillo, madera pulida sin lacar; 5 = Madera burda, tabla o tablón, otro vegetal; 6 = Cemento, gravilla; 7 = Tierra/arena

38. OBSERVE Y ANOTE EL MATERIAL PREDOMINANTE DE LAS PAREDES EXTERIORES DE LA VIVIENDA OCUPADA POR EL HOGAR

1 = Ladrillo o bloque a la vista; 2 = Ladrillo o bloque revocado, pañetado o repellado; 3 = Piedra, madera pulida; 4 = Tapia pisada, adobe; 5 = Bahareque revocado; 6 = Bahareque sin revocar; 7 = Madera burda, tabla, tablón; 8 = Material prefabricado; 9= Guadua, caña, esterilla, otro vegetal; 10= Zinc, tela, lona, cartón, latas, desechos, plástico; 11= Sin paredes

39. ¿Cuántos días por semana permanece habitada esta casa?

40. ¿Cuántas ventanas tiene su vivienda?

41. ¿Las ventanas tienen:

1 = Vidrio, 1=Sí; 2= No	3 = Plastico, 1=Sí;2= No	5 = Cortinas, 1=Sí; 2= No
2 = Angeo, 1=Sí; 2= No	4 = Nada, 1=Sí; 2= No	6 = Otro, 1=Sí; 2= No

42. ¿De cuáles de los siguientes espacios dispone esta vivienda?

1 = Patio, 1=Sí; 2= No
2 = Lote o solar, 1=Sí; 2= No
3 = Antejardín, 1=Sí, 2= No
3 = Garaje o sitio de parqueo, 1=Sí; 2= No
4 = Azotea o terraza, 1=Sí; 2= No
5 = Zonas verdes o zonas de propiedad común, 1=Sí; 2= No

SI CONTESTA "NO" EN JARDÍN O PATIO PASE A PREG. 44

43. ¿Si tiene patio trasero, cuántos árboles, más altos que una persona adulta, hay en el patio?

44. Muestreme su baño, OBSERVE Y ANOTE CON QUÉ TIPO DE SERVICIO SANITARIO CUENTA LA VIVIENDA?

1 = Inodoro conectado al alcantarillado; 2 = Inodoro conectado al pozo séptico; 3 = Inodoro sin conexión; 4 = Letrina (pozo negro, hoyo); 5 = No tienen sanitario

45. ¿Principalmente cómo eliminan la basura de esta vivienda?
ENCUESTADOR: LEA Y REGISTRE CADA UNA DE LAS OPCIONES

1 = La recogen los servicios de aseo; 2 = La recoge un servicio informal (zorra, carreta, etc.);3 = La entierran; 4 = La tiran al río, caño, quebrada o laguna; 5 = La tiran al patio, lote, zanja o baldío; 6 = La queman

46. ¿Con cual de los siguientes servicios públicos, privados o comunales cuenta su vivienda?:
ENCUESTADOR: LEA Y REGISTRE CADA UNA DE LAS OPCIONES

1= Energía eléctrica, 1=Sí; 2= No
2 = Gas natural conectado a red pública? , 1=Sí; 2= No
3 = Acueducto?, 1=Sí; 2= No
4 = Alcantarillado?, 1=Sí; 2= No
5 = Recolección de basuras?, 1=Sí; 2= No
SOLICITE RECIBO PARA ESTRATO Y MARQUE AQUÍ

47. Tiene su vivienda en funcionamiento: *(1=Si, 2= No)*
ENCUESTADOR: LEA Y REGISTRE CADA UNA DE LAS OPCIONES

1= Radio, 1=Sí; 2= No
2= Televisión a color, 1=Sí; 2= No
3 = Lavadora, 1=Sí; 2= No
4 = DVD, 1=Sí; 2= No
5 = Computador, 1=Sí; 2= No
6 = Internet en funcionamiento, 1=Sí; 2= No
7 = Aire acondicionado 1=Sí; 2= No
8 = Ventilador, 1=Sí; 2= No

48. ¿Cuáles son las tres fuentes de información que más utiliza para enterarse de lo que está sucediendo diariamente en el país? Encuestador: NO LEA LAS RESPUESTAS, SOLAMENTE REPORTE LO QUE LA GENTE RESPONDE DE MÁNERA ESPONTÁNEA

1 = Parientes, amigos y vecinos; 2 = Informativo de la comunidad; 3 = Periódico local o de la comunidad; 4 = Periódico nacional; 5 = Radio; 6 = Televisión; 7 = Grupos o asociaciones; 8 = Socios de trabajo o negocios; 9 = Líderes de la comunidad; 10 = Un funcionario del gobierno; 11 = Las ONG; 12 = Internet

V. CONOCIMIENTO SOBRE DENGUE Y EL VECTOR

49. ¿Ha oído hablar de dengue? *(1 = Sí; 2 = No)*

50. Si el encuestado responde SI pregunte: ¿En dónde ha oído hablar de dengue? **SI NO PASE A 55**

Encuestador: NO LEA LAS RESPUESTAS, SOLO REPORTE LO QUE LA GENTE RESPONDE ESPONTANEAMENTE

1 = Parientes, amigos y vecinos, 1=Mencionado; 2= No Mencionado
2 = Informativo de la comunidad, 1=Mencionado; 2= No Mencionado
3 = Periódico local o de la comunidad, 1=Mencionado; 2= No Mencionado
4 = Periódico nacional, 1=Mencionado; 2= No Mencionado
5 = Radio, 1=Mencionado; 2= No Mencionado
6 = Televisión, 1=Mencionado; 2= No Mencionado
7 = Grupos o asociaciones, 1=Mencionado; 2= No Mencionado
8 = Socios de trabajo o negocios, 1=Mencionado; 2= No Mencionado
9 = Líderes de la comunidad, 1=Mencionado; 2= No Mencionado
10 = Un funcionario del gobierno, 1=Mencionado; 2= No Mencionado
11 = Las ONG, 1=Mencionado; 2= No Mencionado
12 = Internet, 1=Mencionado; 2= No Mencionado
13 = Institución de salud, 1=Mencionado; 2= No Mencionado
14= Institución educativa, 1=Mencionado; 2= No Mencionado
15= Otros , Especifique_________________ 1=Mencionado; 2= No Mencionado

51. Hace cuanto recibió la información?

1 = Entre 1 semana y 15 días; **2 =** Hace un mes; **3 =** Entre 2 y 6 meses; **4 =** Mas de 6 meses; **5**= No recuerda

52. ¿Sabe usted cómo se transmite el dengue?

Encuestador: NO LEA LAS RESPUESTAS, SOLO REPORTE LO QUE LA GENTE RESPONDE ESPONTANEAMENTE

1= Por la picadura de un mosquito, 1=Mencionado; 2= No Mencionado
2 = Contacto con alguien enfermo, 1=Mencionado; 2= No Mencionado
3 = Por compartir alimentos, 1=Mencionado; 2= No Mencionado
4 = Por consumir agua y/o alimentos contaminados, 1=Mencionado; 2= No Mencionado
5 = Insectos como pulgas,piojos, moscas, 1=Mencionado; 2= No mencionado
6 = Por la suciedad, 1=Mencionado; 2= No Mencionado
7 = Ratas, pájaros, cerdos, otros animales, 1=Mencionado; 2= No Mencionado
8 = Por la transfusión de sangre, 1=Mencionado; 2= No Mencionado
9= Por compartir artículos de limpieza, 1=Mencionado; 2= No Mencionado
10= No sabe, 1=Mencionado; 2= No Mencionado
11= Otros , Especifique_________________ 1=Mencionado; 2= No Mencionado

53. El dengue se transmite por la picadura de un mosquito, ¿Sabe usted a que hora pica el mosquito?

Encuestador: NO LEA LAS RESPUESTAS, SOLO REPORTE LO QUE LA GENTE RESPONDE ESPONTANEAMENTE

1= En la mañana (hasta las 11 am), 1=Mencionado; 2= No Mencionado
2= Al medio dìa: 12 a 1 pm, 1=Mencionado; 2= No Mencionado
3= En la tarde: 2 a 6 pm, 1=Mencionado; 2= No Mencionado
4= En l anoche: 7 pm en adelante, 1=Mencionado; 2= No Mencionado
5 = Todo el día, 1=Mencionado; 2= No Mencionado
6 = No se, 1=Mencionado; 2= No Mencionado

54. ¿Sabe usted de qué color es el mosquito que transmite el dengue?

1 = Negro; 2 = Café; 3 = Negro con rayas blancas; 9 = Otro

55. ¿Sabe usted en dónde ponen los mosquitos sus huevos o dónde se crían?

Encuestador: NO LEA LAS RESPUESTAS, SOLO REPORTE LO QUE LA GENTE RESPONDE ESPONTANEAMENTE

1=Agua limpia estancada, 1=Mencionado; 2= No Mencionado
2=Agua sucia estancada, 1=Mencionado; 2= No Mencionado
3=Cualquier agua estancada, 1=Mencionado; 2= No Mencionado

4=Sobre ropa mojada, 1=Mencionado; 2= No Mencionado
5=Plantas, 1=Mencionado; 2= No Mencionado
7= No sabe, 1=Mencionado; 2= No Mencionado
9=Otro, cual?(sólo 3 respuestas):_______________________ 1=Mencionado; 2= No Mencionado

56. ¿Alguna vez ha visto larvas en el agua de su vivienda? *1 = Sí; 2 = No; 3 = No se sabe*
Encuestador: mostrar el recipiente con las larvas a la persona encuestada

57. ¿Usted sabe si se puede prevenir el dengue? ***1=** Sí; **2=** No; **3=** No se sabe* — **SI "NO" PASE A 59**

58 ¿Qué pueden hacer las personas en el hogar para evitar el dengue?
Encuestador: NO LEA LAS RESPUESTAS, SOLO REPORTE LO QUE LA GENTE RESPONDE ESPONTANEAMENTE
1 = Lavar las albercas o tanques periódicamente, *1=Mencionado; 2= No Mencionado*
2 = Eliminar todos los recipientes inservibles del patio, jardín o terraza, que puedan recoger agua, *1=Mencionado; 2= No Mencionado*
3 = Tapar los tanques para almacenamiento de agua, *1=Mencionado; 2= No Mencionado*
4 = Usar toldillos impregnados con insecticida, *1=Mencionado; 2= No Mencionado*
5 = Usar repelentes contra insectos*, 1=Mencionado; 2= No Mencionado*
6 = Usar insecticidas para fumigar la casa, *1=Mencionado; 2= No Mencionado*
7 = Evitar aguas estancadas, *1=Mencionado; 2= No Mencionado*
8 = Usar toldillos, *1=Mencionado; 2= No Mencionado*
9 = Usar Angeo o malla en puertas y ventanas, *1=Mencionado; 2= No Mencionado*
10 = Evitar malezas*, 1=Mencionado; 2= No Mencionado*
11 = Evitar compartir alimentos*, 1=Mencionado; 2= No Mencionado*
12 = Evitar contacto con enfermos, *1=Mencionado; 2= No Mencionado*
13 = Evitar compartir artículos de limpieza*, 1=Mencionado; 2= No Mencionado*
14 = Tratar el agua*, 1=Mencionado; 2= No Mencionado*
15 = Manejar adecuadamente desechos*, 1=Mencionado; 2= No Mencionado*
16 = No se puede hacer nada*, 1=Mencionado; 2= No Mencionado*
17 = Usar derivados de petroleo en sitios con aguas sucias o pantanosas*, 1=Mencionado; 2= No Mencionado*
18 = Otro, Explique_____________________*, 1=Mencionado; 2= No Mencionado*
19 = No sabe, *1=Mencionado; 2= No Mencionado*

59. ¿Cuáles manifestaciones (síntomas o signos) le pueden hacer pensar a usted que tiene dengue?
Encuestador: NO LEA LAS RESPUESTAS, SOLO REPORTE LO QUE LA GENTE RESPONDE ESPONTANEAMENTE
1= Fiebre*, 1=Mencionado; 2= No Mencionado*
2= Dolor de cabeza*, 1=Mencionado; 2= No Mencionado*
3= Dolor de huesos*, 1=Mencionado; 2= No Mencionado*
4=Dolor en los músculos*, 1=Mencionado; 2= No Mencionado*
5=Dolor de estómago*, 1=Mencionado; 2= No Mencionado*
6=Dolor en los ojos, 1=Mencionado; 2= No Mencionado
7 = Náuseas y/o Vómito, *1=Mencionado; 2= No Mencionado*
8 = Diarrea, *1=Mencionado; 2= No Mencionado*
9 = Sudoración, *1=Mencionado; 2= No Mencionado*
10 = Puntos rojos en la piel, *1=Mencionado; 2= No Mencionado*
11 = Sangrado en encías, *1=Mencionado; 2= No Mencionado*
12= Sangrado de nariz, *1=Mencionado; 2= No Mencionado*
13 = Debilidad o decaimiento*, 1=Mencionado; 2= No Mencionado*
14 = Otro, Explique_____________________*, 1=Mencionado; 2= No Mencionado*
15= No sabe 1= Mencionado; 2= no mencionado

60. En caso de que usted o alguien en su familia sospechen que tienen dengue ¿Qué hace?
Encuestador: NO LEA LAS RESPUESTAS, SOLO REPORTE LO QUE LA GENTE RESPONDE ESPONTANEAMENTE
1= Toma aspirina, *1=Mencionado; 2= No Mencionado*
2= Se cuida en casa*, 1=Mencionado; 2= No Mencionado*
3= Se automedica*, 1=Mencionado; 2= No Mencionado*
4 = Va al hospital*, 1=Mencionado; 2= No Mencionado*
5 = Consulta en farmacia*, 1=Mencionado; 2= No Mencionado*
6 = No hace nada*, 1=Mencionado; 2= No Mencionado*

61. Usted conoce si existe un tratamiento para el dengue? *1 = Sí; 2 = No; 3 = No se sabe*

Si respondió SI cual? ____

VI. ACTITUDES HACIA EL DENGUE, SU CONTROL Y PREVENCIÓN

62. En su opinión considera que el dengue es un problema para su comunidad? *(1= Sí; 2=No; 3= No sabe)*

63. En su opinión considera que el dengue es un problema para usted? *(1 = Sí; 2=No)*

64. En su opinión, el dengue es una enfermedad grave, moderada o leve?

1 = Grave; 2 = Moderada; 3 = Leve; 4 = Todas; 9 = No sabe

65. Si un vecino se enferma de dengue ¿Usted se puede afectar por este caso? 1= Si; 2= No

66. En su concepto, ¿quién o quiénes son responsables de prevenir el dengue?

Encuestador: NO LEA LAS RESPUESTAS, SOLO REPORTE LO QUE LA GENTE RESPONDE ESPONTANEAMENTE

1= El gobierno, La alcaldía, secretaria de salud, coordinación PIC*, 1 =Mencionado; 2= No Mencionado*
2 = El personal de salud del hospital o de la IPS*, 1=Mencionado; 2= No Mencionado*
3 = Las empresas de servicios públicos, *1=Mencionado; 2= No Mencionado*
4 = Las instituciones de educación*, 1=Mencionado; 2= No Mencionado*
5 = Personal de otra entidad, *1=Mencionado; 2= No Mencionado*
6 = Personas de la comunidad*, 1=Mencionado; 2= No Mencionado*
7 = Uno mismo*, 1=Mencionado; 2= No Mencionado*
8 = No sabe*, 1=Mencionado; 2= No Mencionado*

67. ¿Qué debería hacer el gobierno para mejorar la prevención del dengue?

Encuestador: NO LEA LAS RESPUESTAS, SOLO REPORTE LO QUE LA GENTE RESPONDE ESPONTANEAMENTE

1 = Verifica depósitos de agua, 1=Mencionado; 2= No Mencionado
2 = Agrega químicos en el agua, 1=Mencionado; 2= No Mencionado
3 = Fumiga al interior de la casa, 1=Mencionado; 2= No Mencionado
4 = Fumiga al exterior de la casa , 1=Mencionado; 2= No Mencionado
5 = Suministra cubiertas para los recipientes, 1=Mencionado; 2= No Mencionado
6 = Suministra peces para tener en el agua, 1=Mencionado; 2= No Mencionado
7 = Corta plantas, 1=Mencionado; 2= No Mencionado
8 = Educa a las personas, 1=Mencionado; 2= No Mencionado
9 = Otro especifique_____________1=Mencionado; 2= No Mencionado
10 = Nada, 1=Mencionado; 2= No Mencionado

VII. PRÁCTICAS EN DENGUE Y EL VECTOR

68. ¿Qué hace usted en casa para reducir la incomodidad ocasionada por los mosquitos?

Encuestador: LEA Y MARQUE CADA UNA DE LAS RESPUESTAS

1 = Nada, 1=Sí; 2= No
2 = Fumigación al interior de la casa, 1=Sí; 2= No
3 = Limpieza de basura, 1=Sí; 2= No
4 = Cubrimiento de depósitos de agua, 1=Sí; 2= No
5 = Agrega químicos en el agua, 1=Sí; 2= No
6 = Tiene animales en el agua (peces, tortugas), 1=Sí; 2= No
7 = Matar mosquitos, por ej., con químicos, plaquitas o bobina de mosquito, 1=Sí; 2= No
8 = Protección personal con repelentes, 1=Sí; 2= No
9 = Uso de angeos o toldillos, 1=Sí; 2= No
10 = Educa a otros miembros de la familia y/o vecinos sobre las medidas para disminuir los mosquitos 1=Sí; 2= No
11= Lavar las albercas o tanques periódicamente, *1= Si 2=No*
13= Eliminar todos los recipientes inservibles del patio, jardín o terraza, que puedan recoger agua, 1= Si 2=No
12=Otros *1= Si 2=No*

SI NO FUMIGA O MATA MOSQUITOS PASE A 71. SI MATA MOSQUITOS, PASE A 70

69. Si fumiga al interior de la casa, ¿Cada cuanto lo hace?

(1 = Todos los días; 2 = una vez por semana; 3 = Dia de por medio; 4 = Cada semana; 5 = Cada 15 días; 6 = Cada mes; 7 = Mas del mes; 8 = No sabe)

70. Que tipo de insecticida utiliza?

Líquido, 1=Sí; 2= No Cual: ______________________________
Aerosol, 1=Sí; 2= No Cual: ______________________________
Plaquitas, 1=Sí; 2= No Cual: ______________________________
Espiral, 1=Sí; 2= No Cual: ______________________________
Otro, 1=Sí; 2= No Cual:

71. ¿Cuánto dinero gasta al mes para reducir la incomodidad ocasionada por los mosquitos?

INTRODUZCA LA CIFRA REPORTADA POR EL ENCUESTADO

72. ¿Usted almacena agua? *1= Sí; 2= No*

SI CONTESTA QUE NO ASEGURESE DE QUE NO TENGA AGUA EN LA ALBERCA, O EN CANECAS **SI NO PASE A 76**

73. ¿Para qué almacena usted el agua? (Encuestador: preguntar uno por uno y registrar)

1=Para lavar la ropa, *1=Sí; 2= No*

2=Para limpiar la casa, *1=Sí; 2= No*

3=Para consumo humano, *1=Sí; 2= No*

9=Otras, especifique: _______________*1=Sí; 2= No: 9=no especifica otras.*

74. Si usted almacena agua, con qué frecuencia vacía cada uno de los siguientes recipientes?

Encuestador: preguntar la frecuencia para cada tipo de recipiente)

(1=todos los días; 2=día de por medio; 3=cada semana; 4=cada 15 días 5=cada mes; 6=nunca; 9= No tiene)

1. Recipientes grandes (más de 200 litros)
2. Recipientes medianos (canecas metálicas, tanques de cemento etc.)
3. Recipientes pequeños (baldes, jarras etc.)

75. ¿Cuál es su principal fuente de abastecimiento de agua para almacenar?

1=Acueducto; 2=Pozo con bomba; 3= Pozo sin bomba, jagüey; 4=Rio, quebrada, manantial, nacimiento; 8 = Agua lluvia; 9=Otros (especifique: ________________________)

76. ¿Dónde desecha el material inservible como: botellas, llantas, latas, tarros, envaces, cascarones de huevos, juguetes etc.? (espere respuesta)

1 = Carro recolector, 1=Mencionado; 2= No Mencionado

2 = Almacena en la vivienda a la intemperie, 1=Mencionado; 2= No Mencionado

3 = Entierra, 1=Mencionado; 2= No Mencionado

4 = Otra, 1=Mencionado; 2= No Mencionado

5 = No lo desecha, 1=Mencionado; 2= No Mencionado

77. ¿Cada cuánto elimina el material inservible? (lea opciones)

1 = Cada vez que pase el carro recolector; 2 = Cada semana; 3 = Cada dos semanas; 4 = Cada mes; 5 = Mas del mes; 6 = Nunca elimina inservibles

VIII. ACCIONES REALIZADAS POR LAS ENTIDADES PÚBLICAS

78. ¿Hace cuánto recibió la última visita de un técnico de vectores?

1= En el último mes; 2= Hace 2 o 6 meses; 3= Hace 7 a 12 meses; 4= Hace más de 1 año; 5= No recuerda/nunca

79. En la última visita, ¿le dieron alguna instrucción sobre cómo evitar la reproducción del mosquito? (*1 = Sí; 2 = No; 3 = No se sabe)*

80 ¿Alguna vez ha recibido instrucciones o materiales (ej, material impreso, químicos, control biológico) para prevenir el dengue? *(1 = Sí, 2 = No)*

Si la respuesta es SI, especifique____

81. Ha participado en actividades de prevención y/o control del dengue? *1 = Sí; 2 = No*

82. ¿Existe algún esfuerzo comunitario para limpiar el ambiente? *(1 = Sí; 2 = No; 3 = No se sabe)* **SI "NO" PASE A 84**

83. Si la respuesta es SI, ¿Qué hace la comunidad para eliminar los mosquitos de dengue?

1= Elimina criaderos, 2= Visita hogares para asegurar la implementación del programa, 3=Otros; especifique_______________________

84. ¿Qué medidas de control realiza el gobierno contra los mosquitos?

Encuestador: NO lea las respuestas sólo indique lo que la persona encuestada responde de manera espontánea

1 = Verifica depósitos de agua, 1=Mencionado; 2= No Mencionado

2 = Agrega químicos en el agua, 1=Mencionado; 2= No Mencionado

3 = Fumiga al interior de la casa, 1=Mencionado; 2= No Mencionado

4 = Educa a las personas, 1=Mencionado; 2= No Mencionado

5 = Suministra cubiertas para los recipientes, 1=Mencionado; 2= No Mencionado

6 = Suministra peces para tener en el agua, 1=Mencionado; 2= No Mencionado

7 = Fumigación al exterior de la casa, 1=Mencionado; 2= No Mencionado

8 = Corta plantas, 1=Mencionado; 2= No Mencionado

9 = Otro especifique____________1=Mencionado; 2= No Mencionado

13 = Nada, 1=Mencionado; 2= No Mencionado

14= No Sabe, 1=Mencionado; 2= No Mencionado
